# Prevalence of preoperative deep vein thrombosis in long bone fractures of lower limbs: a systematic review and meta-analysis

**DOI:** 10.1186/s10195-023-00699-2

**Published:** 2023-05-08

**Authors:** Yaowen Hu, Liwen Zhu, Xuan Tian, Fangfang Duan

**Affiliations:** 1 Beijing Jishuitan Hospital, The Fourth Clinical Medical College, Peking University Health Science Center, Beijing, China; 2 Department of Vascular Surgery, Beijing Jishuitan Hospital, Beijing, China; 3 Clinical Epidemiology Research Center, Beijing Jishuitan Hospital, Beijing, China

**Keywords:** Deep vein thrombosis, Prevalence, Long bone, Preoperative, Lower extremity fracture

## Abstract

**Background:**

There is a paucity of data regarding the prevalence of preoperative deep vein thrombosis (DVT) in patients with long bone (including femur, tibia and fibula) fractures of the lower limbs. We performed a meta-analysis to address the issue.

**Methods:**

Electronic databases, including PubMed, EMBASE, the Web of Science, the Cochrane Library, the VIP database, CNKI, and the Wanfang database, were systematic searched for original articles that reported the prevalence of preoperative DVT in long bone fractures of the lower limbs from January 2016 to September 2021. The prevalence of preoperative DVT was pooled using random-effects models, and subgroups were established according to study type, detection method, sample size and fracture site.

**Results:**

Twenty-three articles reporting on 18,119 patients were eligible. The overall pooled preoperative DVT prevalence was 24.1% (95% CI 19.3–28.8%). In different subgroups, the preoperative DVT prevalences were 18.2–27.3%, 15.2–28.6%, 23.1–24.9%, 18.2–26.0% and 23.2–23.4% for different study designs, sample sizes, age groups, detection methods and fracture sites, respectively.

**Conclusions:**

Despite the heterogeneity among studies, this systematic review suggests that the prevalence of preoperative DVT, which may seriously affect the prognosis of patients, is high. Therefore, greater efforts should be devoted to the improvement of screening and prevention strategies for preoperative DVT in lower-extremity long bone fractures.

*Level of Evidence:* Level III.

*Trial Registration* The study was registered in the International Prospective Register of Systematic Reviews (PROSPERO) database with the registration number CRD42022324706.

## Introduction

Fractures of the long bones of the lower extremities, including the femur, tibia and fibula, are often accompanied by high-energy injuries. Fracture ends can cause venous endothelial damage, blood is in a post-traumatic hypercoagulable state, and bone traction and long-term bed rest-immobilization can result in slow blood flow, all of which meet the conditions for thrombosis. Therefore, deep vein thrombosis (DVT) is common in patients with fractures of long bones of the lower extremities. It has been reported that DVT in periprosthetic and lower extremity fractures accounts for more than 95% of DVT patients in traumatic orthopedics, while that in upper extremity fractures is rare, with an overall incidence of 0.69% [1–3]. DVT can lead to prolonged hospitalization and increased hospitalization expenses [4]. Also, subsequent post-thrombotic syndrome (PTS) and pulmonary embolism (PE) may seriously affect the patient’s life quality and even lead to their death [5, 6].

Perioperative DVT is divided into preoperative and postoperative DVT according to the occurrence time. Besides the consequences mentioned above, preoperative DVT may cause a delay to surgery, shifting it from the optimal surgical timing and thus affecting the outcomes; more seriously, if a thrombus is not detected in time preoperatively, orthopedic surgery will cause it to break off, leading to PTS, PE and other adverse outcomes [7]. However, the existing guidelines do not distinguish between preoperative and postoperative DVT in terms of screening and diagnostic strategies. For patients with lower extremity fractures, routine venous ultrasound is recommended [8]. The prevention and management of postoperative DVT has been given more attention than those of preoperative DVT. We believe that the clarification of the prevalence of preoperative DVT in patients with long bone fractures of the lower extremities will help improve the standardization of preoperative DVT prevention, screening, diagnosis and treatment, and is conducive to the rational allocation of health resources.

However, only a small part of the literature focuses on the prevalence of preoperative DVT, and the results show a large heterogeneity. The prevalence of preoperative DVT in patients with proximal femoral fractures was reported to be 52.50% [9], whereas studies from Hong Kong revealed that the prevalence of preoperative DVT after hip fracture in elderly Chinese patients was low (5.3%) without thromboprophylaxis. From their perspective, routine venous thromboprophylaxis in those patients was not recommended [10]. One study noted a preoperative DVT prevalence of 43.92% for tibial plateau fractures [11], whereas another study reported a prevalence of only 16.3% [12]. In conclusion, there are conflicting statements regarding preoperative DVT prevalence and prophylaxis.

This systematic review aimed to investigate the prevalence of preoperative DVT in patients with long bone fractures of the lower extremities, providing a basis for clarifying the disease burden of preoperative DVT and developing reasonable screening strategies and preventative measures.

## Methods

### Search strategy

We searched PubMed, EMBASE, the Web of Science, the Cochrane Library, the VIP database, CNKI, and the Wanfang database for articles reporting the prevalence of preoperative DVT in patients with long bone fractures of the lower extremities published between January 2016 and September 2021, using the following search terms: (Femoral Fractures OR Tibial Fractures OR Fibular fractures) AND Venous Thrombosis. A comprehensive search of the literature was performed to identify all relevant studies. The references of the included studies were searched manually. The study was registered in the International Prospective Register of Systematic Reviews (PROSPERO) database with the registration number CRD42022324706.

### Selection criteria

#### Inclusion criteria

The inclusion criteria were as follows: (i) studies containing sufficient information on the prevalence of preoperative DVT in long bone fractures of the lower extremities, which include femoral neck, femoral shaft, intertrochanteric, subtrochanteric, tibial plateau, tibial shaft and fibula fractures; (ii) clinical trials, case–control studies and cohort studies; (iii) the study population included patients with preoperative DVT determined by color Doppler ultrasonography, duplex ultrasonography or venography following long bone (femur, tibia or fibula) fractures of the lower extremities; (iv) studies with a score of  ≥ 6 on the Newcastle–Ottawa scale (NOS).

#### Exclusion criteria

The exclusion criteria were as follows: (i) reviews, case reports, conference papers or animal studies; (ii) studies with incomplete data that could not be combined, duplicate publications, literature for which the full text was not available, or case–control studies from which the total number of patients (needed to calculate the DVT prevalence) could not be obtained; (iii) studies including patients with pathological fractures (fractures caused by bone tuberculosis, osteomyelitis, bone tumors, osteoporotic fractures, etc.); (iv) studies reporting the preoperative DVT prevalence in fractures at sites other than lower-extremity long bones that were not discussed separately, making it impossible to extract the required data; (v) the time order of DVT and surgery was not clearly defined; (vi) the subject was venous thromboembolism (VTE, divided into DVT and PE), and the DVT prevalence was unclear.

### Article screening

The retrieved publications were managed using EndNote X9 software. Firstly, duplicates were eliminated by the software based on information such as title, author, year of publication, and journal. After that, two researchers independently read the titles and abstracts for initial screening, and then the full texts were downloaded so that they could be thoroughly reviewed. Any inconsistency in the process was decided by the third researcher.

### Quality assessment

The included studies were independently evaluated in terms of study design by two investigators using the Newcastle–Ottawa scale [13]. Studies with NOS scores ≥ 6 were included in the subsequent analysis.

### Data extraction

The extraction of data from the eligible studies was performed independently by two researchers. A third researcher decided in the case of disagreement. An Excel table was established to collect relevant information including, but not limited to, the first author, year of publication, study design, country, study duration, sample size, NOS score, locations of fractures, DVT detection method and preoperative DVT prevalence.

### Statistical analysis

All statistical evaluations were made using StataSE 15 (64 bit). The prevalence of preoperative DVT was calculated as the simple rate and measured with 95% confidence intervals (CIs). Interstudy heterogeneity was tested using the* I*^2^ test, and when *P* > 0.1 and *I*^2^ ≤ 50%, the heterogeneity was considered statistically insignificant and a fixed-effects model was applied. Otherwise, a random-effects model was used. Subgroups were divided according to study type, testing modality, sample size, and fracture site, and the prevalence was estimated for different subgroups. Begg’s statistical test was performed to assess the publication bias.

## Results

### Study selection

**Fig. 1:**
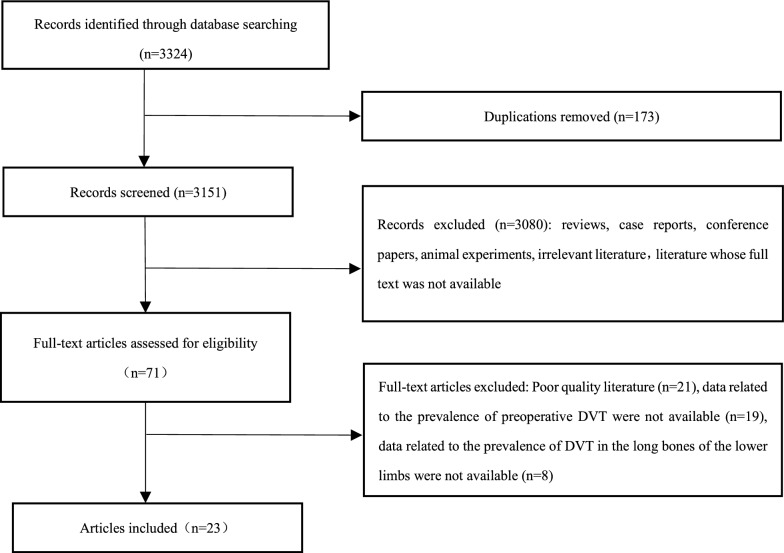
Flow diagram of study selection

Of 3324 articles, 71 were eligible for full-text screening, and 23 original studies ultimately met the selection criteria (Fig. 1).

### Study characteristics and quality assessment

For the 23 included studies, the years of publication ranged from 2016 to 2021, and, considered together, the durations of the studies covered the period from January 2010 to June 2020. The total number of lower extremity long bone fractures was 18,119, and the sample size in each study ranged from 119 to 7177. Eighteen studies had a NOS score of 7 and five studies had a NOS score of 6 (Table 1).

**Table 1:** Characteristics of the included studies and detailed information on the patients

Study no.	Author	Year of publication	Study design	Country	Study duration	Sample size	Male patients	Age (year)	NOS score	Locations of fracture	DVT detection method	Prevalence of preoperative DVT %	Patients with long bone fractures of lower extremities	Prevalence of preoperative DVT in long bone fractures of lower extremities %
1	Shin et al. [14]	2016	Retrospective cohort study	Korea	December 2010–August 2014	208	62	75.9 ± 9.7 (range 27–97)	7	Femoral neck, intertrochanteric, subtrochanteric	Indirect MDCT venography	7.69	208	7.69
2	Song et al. [15]	2016	Retrospective cohort study	China	September 2010–July 2014	119	42	75.2 ± 9.7 (range 47–92)	6	Femoral neck	Ascending venography	29.40	119	29.40
3	Wang et al. [16]	2018	Retrospective case–control study	China	September 2014–September 2017	1825	879	62.8 ± 19.5(range 18–102)	6	Femoral neck, intertrochanteric, femoral shaft, tibial plateau, tibial shaft	Duplex ultrasonography	30	1643	31.65
4	Wang et al. [17]	2018	Nested case–control study	China	January 2016–June 2017	248	108	71.8 ± 13.7 (range 45–96)	7	Femoral neck, trochanteric	Doppler ultrasonography	11.70	248	11.70
5	Xia et al. [18]	2018	Retrospective case–control study	China	January 2014–March 2017	301	95	76.45	6	Femoral neck	Doppler ultrasonography	18.90	301	18.90
6	Zhang et al. [19]	2018	Retrospective case–control study	China	July 2014–October 2016	463	175	72.86 ± 13.79 (range 19–102)	7	Intertrochanteric, subtrochanteric, femoral neck	Doppler ultrasonography	34.98	463	34.98
7	Fei et al. [11]	2019	Retrospective case–control study	China	September 2014–December 2017	148	90	47. 2 ± 13. 1 (range 19–83)	7	Tibial plateau	Color Doppler ultrasound	43.92	148	43.92
8	Li et al. [20]	2019	Retrospective case–control study	China	September 2014–February 2018	180	114	47.6(range 16–83)	7	Tibia, fibula	Color Doppler ultrasound	21.7	180	21.70
9	Fei et al. [21]	2020	Retrospective case–control study	China	July 2015–October 2017	218	85	76.0 ± 11.9 (range 32–102)	7	Intertrochanteric	Color Doppler ultrasound	37.6	218	37.60
10	Li et al. [22]	2020	Retrospective case–control study	China	June 2014–September 2018	485	196	74. 6 (range 16–102)	7	Intertrochanteric	Color Doppler ultrasound	36.5	485	36.50
11	Wei et al. [23]	2020	Retrospective case–control study	China	January 2017–December 2018	242	99	69.1 (range 15–96)	7	Femoral neck, intertrochanteric	Color Doppler ultrasound	24.0	242	24
12	Feng et al. [24]	2020	Retrospective case–control study	China	January 2012–December 2018	273	72	78 ± 11	7	Femoral neck, intertrochanteric, subtrochanteric, proximal femoral shaft	Ultrasound (unspecified type)	5.60	273	5.60
13	Fu et al. [25]	2020	Retrospective case–control study	China	July 2016–December 2018	228	78	71.28 ± 13.47	6	Femoral neck	Ultrasound (unspecified type)	32	228	32
14	Li et al. [26]	2020	Retrospective case–control study	China	October 2014–December 2018	140	85	47.33 ± 12.90	6	Tibial plateau	Duplex ultrasonography	36.43	140	36.43
15	Liu et al. [12]	2020	Retrospective cohort study	China	May 2018–December 2019	1179	742	45.6 ± 13.6 (range,18–82)	7	Tibial plateau	Duplex ultrasonography	16.30	1179	16.30
16	Ma et al. [27]	2020	Prospective cohort study	China	December 2014–October 2017	918	672	44.6 ± 14.5 (range,18–90)	7	Tibial shaft	Duplex ultrasonography	13.30	918	13.30
17	Zhang et al. [9]	2020	Prospective cohort study	China	October 2018–June 2020	160	59	58.82 ± 16.01	7	Distal femur	Duplex ultrasonography	52.50	160	52.50
18	Zuo et al. [28]	2020	Retrospective cohort study	China	January 2017–December 2019	578	217	76.6 ± 8.7 (range 60–102)	7	intertrochanteric	Doppler ultrasonography	20.10	578	20.10
19	Bai et al. [29]	2021	Retrospective case–control study	China	July 2017–October 2019	264	144	50.69 ± 12.72(range 14–86)	7	Tibial plateau	Color Doppler ultrasound	39.0	264	39.00
20	Chang et al. [30]	2021	Retrospective cohort study	China	July 2014–November 2018	11,891	5330	61.4 + 8.29 (range 18–93)	7	Femoral neck, intertrochanteric, proximal femur, femoral shaft, tibial, fibula	Color Doppler ultrasound	4.86	7177	5.10
21	Fan et al. [31]	2021	Retrospective case–control study	China	January 2010–December 2019	788	273	78.68 ± 7.89 (range 60–113)	7	Intertrochanteric	Color Doppler ultrasound	20.81	788	20.81
22	Niu et al. [32]	2021	Retrospective case–control study	China	January 2016–October 2019	980	310	72.5 ± 8.5 (range 60–96)	7	Femoral neck	Duplex ultrasonography	6.80	980	6.80
23	Zhu et al. [33]	2021	Prospective cohort study	China	October 2014–December 2018	1179	742	45.6 ± 13.6 (range, 18–82)	7	Tibial plateau	Duplex ultrasonography	16.30	1179	16.30

### Prevalence of preoperative DVT in long bone fractures of the lower extremity

**Fig. 2:**
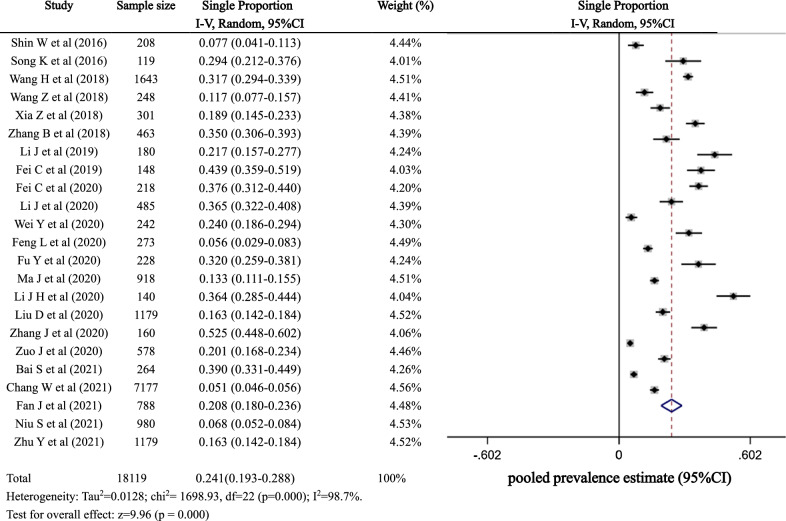
Forest plot of preoperative DVT prevalence in long bone fractures of the lower extremity

In the included literature, the reported preoperative DVT prevalence ranged from 5.10% to 52.50%, with significant heterogeneity between studies (*p* < 0.01, *I*^2^ = 98.7%), so a random-effects model was used. The pooled prevalence of preoperative DVT in long bone fractures of the lower extremities was 24.1% (95% CI 19.3–28.8%), as detailed in Fig. 2.

**Fig. 3:**
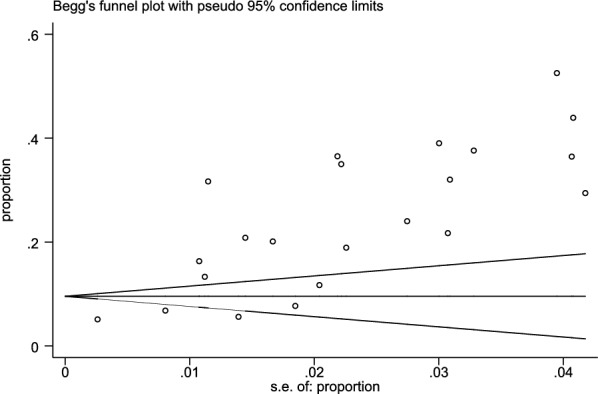
Funnel plot showing no significant publication bias

Begg’s test was performed on these 23 publications and found no significant publication bias (*p* > 0.05), as shown in Fig. 3.

### Subgroup analysis

#### Study design

The 23 included papers were classified into different subgroups according to study design. Six were retrospective cohort studies, one was a nested case–control study, 13 were retrospective case–control studies, and three were prospective cohort studies.

**Fig. 4:**
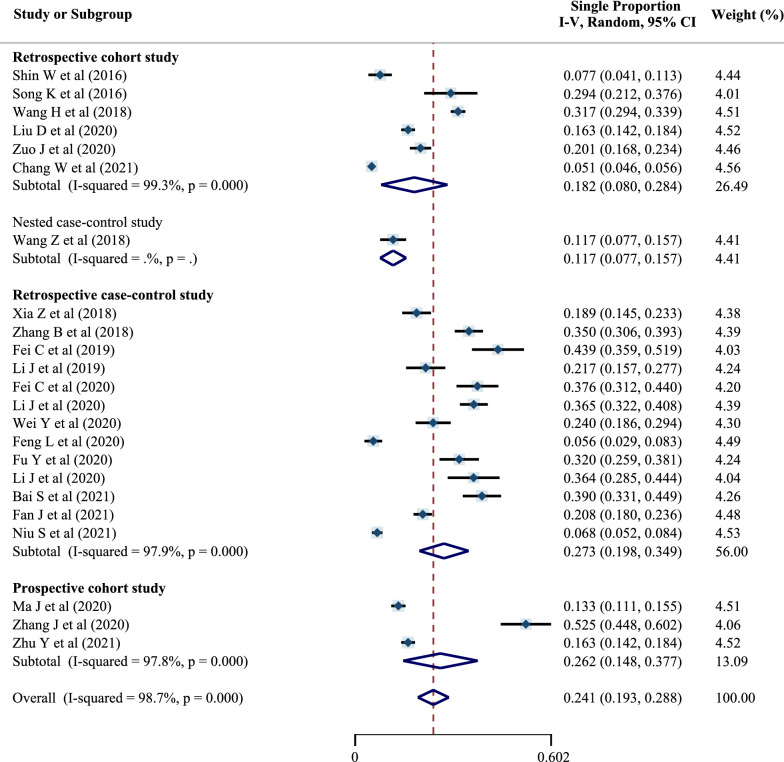
Forest plot of preoperative DVT prevalence in different study-type subgroups

The pooled prevalence was 18.2% (95% CI 8.0–28.4%) among retrospective cohort studies, 27.3% (95% CI 19.8–34.9%) among retrospective case–control studies and 26.2% (95% CI 14.8–37.7%) among prospective cohort studies (Fig. 4). A random-effects model was used due to significant heterogeneity.

#### Sample size

The 23 included papers were divided up according to the sample size. Fifteen had a sample size  of ≤ 500, four had a sample size of 500–1000, and four had a sample size  ≥ 1000.

**Fig. 5:**
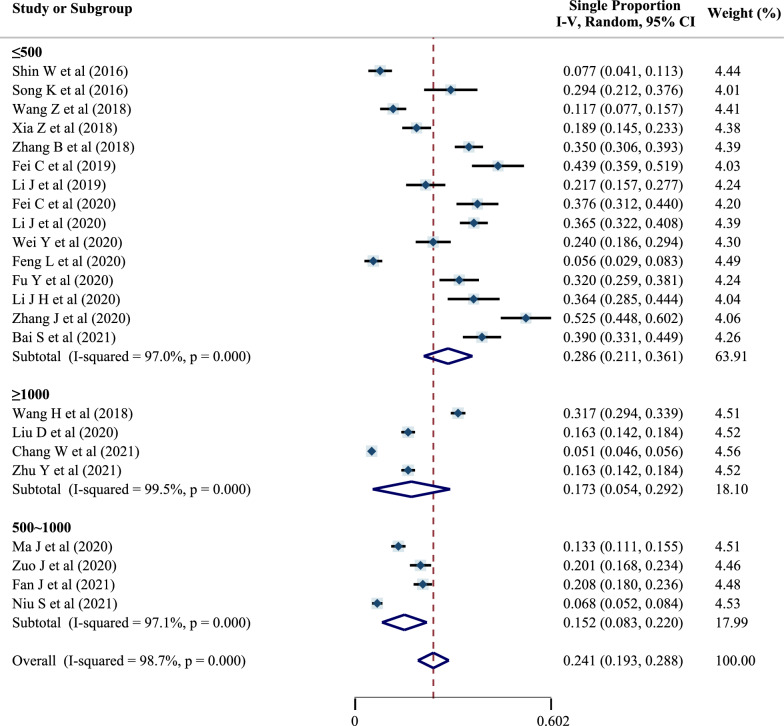
Forest plot of preoperative DVT prevalence in different sample-size subgroups

The pooled prevalence was 28.6% (95% CI 21.1–36.1%) in the subgroup with a sample size of ≤ 500, 15.2% (95% CI 8.3–22.0%) in the subgroup with a sample size of 500–1000, and 17.3% (95% CI 5.4–29.2%) in the subgroup with a sample size of  ≥ 1000, as shown in Fig. 5. The *I*^2^ test revealed significant heterogeneity, so a random-effects model was used for meta-analysis.

#### Mean age

Twenty-one publications reported relevant information on the patients’ mean age: the mean age in 15 studies was  ≥ 50 years old, and that in six studies was  < 50 years old.

**Fig. 6:**
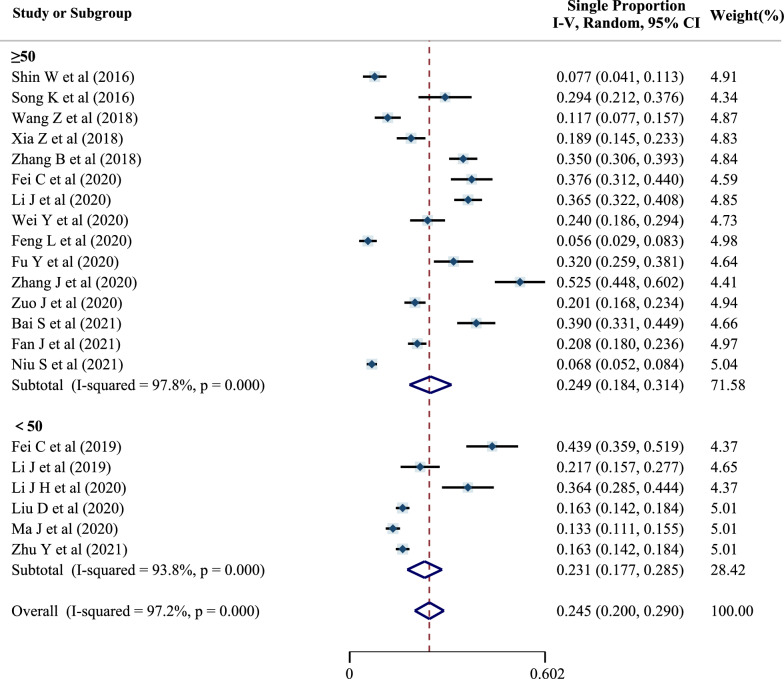
Forest plot of preoperative DVT prevalence in different mean-age subgroups

The preoperative DVT prevalence was 24.9% (95% CI 18.4–31.4%) for patients with a mean age of ≥ 50 years old and 23.1% (95% CI 17.7–28.5%) for patients with mean age of  < 50 years old (Fig. 6). Taking into account the significant heterogeneity, a random-effects model was used. The chi-square test was adopted to assess whether there was a statistical difference in DVT prevalence between the two subgroups (Pearson chi^2^ = 3.9219, *P* = 0.048).

#### DVT detection method

Subgroups were established according to the detection method: 12 used Doppler ultrasound, seven used duplex ultrasound, and two used venography. The remaining two did not specify the type of ultrasound used.

**Fig. 7:**
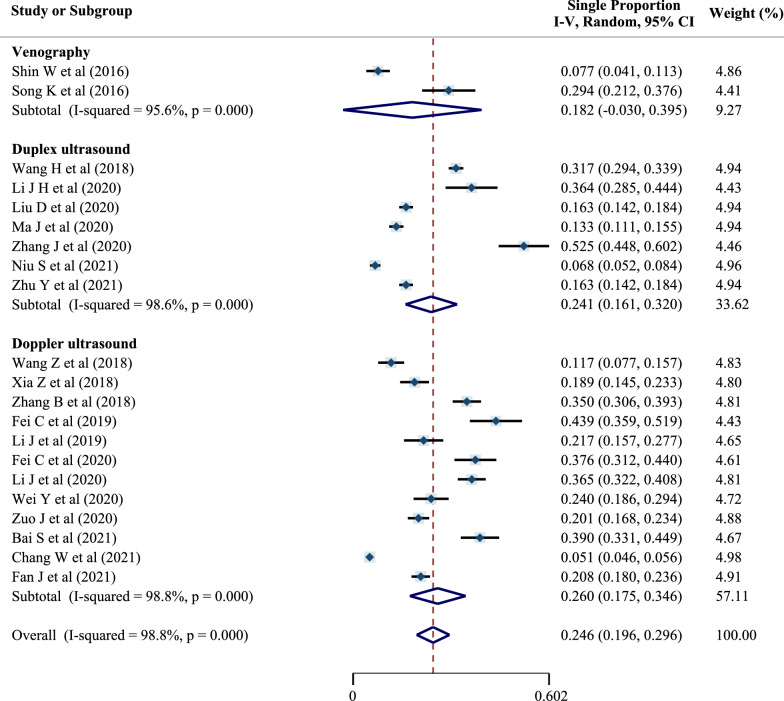
Forest plot of preoperative DVT prevalence in different detection-method subgroups

The preoperative DVT prevalence differed by detection method (18.2% [95% CI: − 3.0 to 39.5%] for the venography subgroup, 24.1% [95% CI 16.1–32.0%] for the duplex ultrasound subgroup, and 26.0% [95% CI 17.5–34.6%] for the subgroup that used Doppler ultrasound), as shown in Fig. 7.

#### Fracture site

Sixteen studies could be used to analyze the preoperative DVT prevalence of femoral fractures, and nine could be used to analyze that of tibiofibular fractures (two of them—those of Wang et al. [16] and Chang et al. [30]—included data related to both femoral and tibiofibular fractures), as detailed in Tables 2 and 3.

**Table 2:** Prevalence of preoperative DVT in patients with femoral fractures

Study no.	Year	Study	*n*	*r*
1	2016	Shin et al. [14]	208	0.0769
2	2016	Song et al. [15]	119	0.2940
3	2018	Wang et al. [16]	1239	0.3406
4	2018	Wang et al. [17]	248	0.1170
5	2018	Xia et al. [18]	301	0.1890
6	2018	Zhang et al. [19]	463	0.3498
7	2020	Fei et al. [21]	218	0.3760
8	2020	Li et al. [22]	485	0.3650
9	2020	Wei et al. [23]	242	0.2400
10	2020	Feng et al. [24]	273	0.0560
11	2020	Fu et al. [25]	228	0.3200
12	2020	Zhang et al. [9]	160	0.5250
13	2020	Zuo et al. [28]	578	0.2010
14	2021	Chang et al. [30]	5216	0.0620
15	2021	Fan et al. [31]	788	0.2081
16	2021	Niu et al. [32]	980	0.0680

**Table 3:** Prevalence of preoperative DVT in patients with tibiofibular fractures

Study no.	Year	Study	*n*	*r*
1	2018	Wang et al. [16]	404	0.2426
2	2019	Li et al. [20]	180	0.2170
3	2019	Fei et al. [11]	148	0.4390
4	2020	Ma et al. [27]	918	0.1330
5	2020	Li et al. [26]	140	0.3643
6	2020	Liu et al. [12]	1179	0.1630
7	2021	Bai et al. [29]	264	0.3900
8	2021	Chang et al. [30]	1961	0.0209
9	2021	Zhu et al. [33]	1179	0.1630

**Fig. 8:**
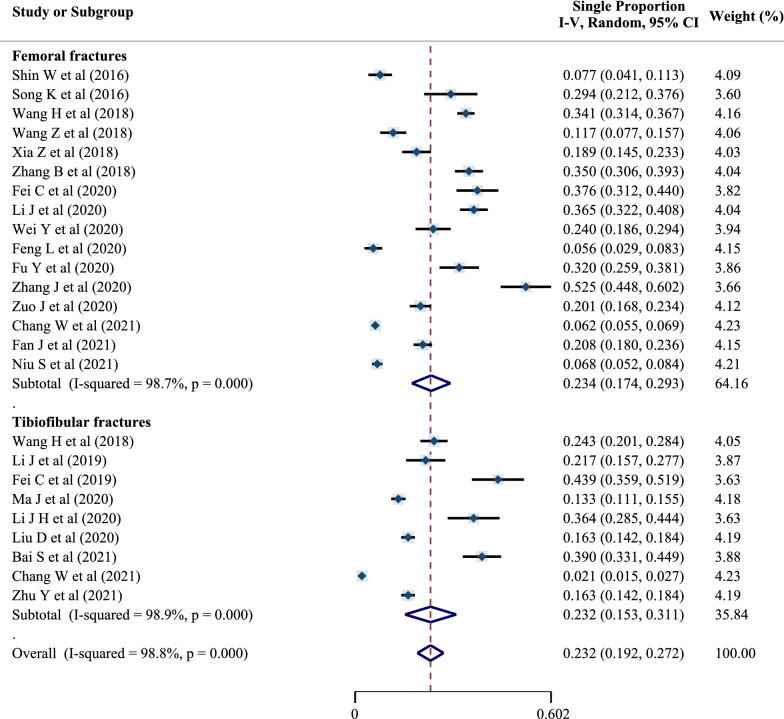
Forest plot of preoperative DVT prevalence in different fracture sites

The preoperative DVT prevalence was 23.4% (95% CI 17.4–29.3%) for patients with femoral fractures and 23.2% (95% CI 15.3–31.1%) for patients with tibiofibular fractures, as shown in Fig. 8. There was significant heterogeneity, so a random-effects model was used.

## Discussion

Based on our research, the pooled prevalence of preoperative DVT in lower-extremity long bone fractures was up to 24.1%. The results of these analyses suggest that the high prevalence of preoperative DVT, which is closely related to the patient’s prognosis, should be given more attention in clinical practice, and the differences in results between distinct subgroups indicate the influences of different factors in the formation of DVT.

Our study reported a higher preoperative DVT prevalence in patients with femoral fractures compared to tibiofibular fractures, which is generally consistent with the results of previous articles. Some studies have revealed that preoperative DVT prevalence was higher in femoral shaft fractures, proximal femoral fractures, and distal femoral fractures than in tibial plateau fractures and tibiofibular fractures [1, 34, 35]. Femoral fractures are usually due to higher energy, and vascular distribution in the thigh is dense, leading to severe endovascular injury and more bleeding, which contributes to a higher prevalence of DVT [1]. The classification method for fracture sites varied among the current studies, so our article is unable to provide more detailed categorization of the fracture sites. Our data indicate that DVT detection should be prioritized in patients with femoral fractures in the future due to the high prevalence of DVT in such fractures.

The prevalence of preoperative DVT in lower extremity fractures is higher in older adults than in younger adults, and the difference in DVT prevalence between these two subgroups was significant according to the results of a chi-square test. This finding suggests that advanced age may be an independent risk factor for the occurrence of preoperative DVT in long bone fractures of the lower extremities, which has been confirmed in previous studies [9, 17, 19]. Patients of advanced age are in a hypercoagulable state due to prolonged post-injury inactivity, and the poor vascular elasticity further increases the risk of DVT. Of note, eight of the included studies reported the use of prophylactic anticoagulation therapy (e.g., low-molecular-weight heparin) prior to the detection of DVT among patients with a mean age of  ≥ 50 years. The use of antithrombotic medication could be a confounding factor that potentially influenced the results for different age subgroups. However, we cannot currently tell whether the age factor was influenced—and, if so, the degree of influence—due to a lack of evidence.

The sensitivity and specificity of DVT detection depended on the detection method. As previously noted [36], compared with venography, the sensitivity of duplex ultrasound for DVT detection was 92.1% and its specificity was 94.0%, whereas the sensitivity of the combined color Doppler ultrasound technique was 81.7% (77.4–85.5%) and its specificity was 92.7% (89.7–95.1%). However, the detection rate was lower in the color Doppler ultrasound subgroup than in the duplex ultrasound subgroup in our study. The workload of ultrasound examiners and the operational experience of physicians vary depending on the hospital, with some studies showing higher diagnostic consistency among experienced sonographers and a decrease in diagnostic consistency with less experience in hip ultrasound using Graf’s method [37]. The difference stated above may lead to differences in false-negative rates, thus affecting diagnostic accuracy. Differences in the location and time of ultrasound scanning between different studies (including scanning only the fractured lower limbs or scanning both lower limbs; performing one scan or multiple scans; etc.) may also be the source of error. The convenience and non-invasiveness of ultrasonography make it a first-line detection method for DVT despite its lower sensitivity compared to venography. In our analysis, the DVT prevalence was lower in the venography subgroup than in the two ultrasound subgroups, which does not seem to be consistent with the high sensitivity of venography. Shin [14] and colleagues performed venography only in patients, with a delay of over 24 h from the time of injury to surgery, which could lead to missed detection, while Song [15] and colleagues excluded patients with a history of VTE, which could lead to a lower DVT prevalence. The prevalence of preoperative DVT varied widely among the included studies. Chang [30] reported a DVT prevalence of only 5.10%, whereas in a study by Zhang [9], the prevalence of DVT was up to 52.5%. According to Zhang [9], DUS was conducted immediately after admission, but in the study of Chang [30], patients were all given subcutaneous low-molecular heparin injections upon admission, after which ultrasound was performed, implying that some patients who had formed a DVT might have been on heparin therapy before detection, which may explain the decrease in the prevalence of DVT. Among all the included studies, the timing of DVT detection was the first day after admission in four papers; 1 day before surgery in eight papers, seven of which mentioned prophylactic anticoagulation before testing; seven papers described the timing of testing only as "after admission" or "before surgery;" and the remaining three did not specify the timing of the study. The different timings of testing and anticoagulation strategies could lead to reduced comparability across studies. The existing guidelines, however, do not specify the timing and scanning area of preoperative DVT testing [8]. Our study suggests that preoperative DVT detection strategies need to be more standardized and detailed, which would provide guidance for the improvement of guidelines for perioperative DVT prevention and diagnosis.

The prevalence of DVT varied with the population characteristics. The DVT prevalence was lowest in the 500–1000 patient subgroup, with the lowest prevalence reported by Ma [27] and Niu [32]. The mean age of the sample in Ma’s [27] study was 44.6 years, which was much lower than the mean age across all the included literature; Niu's [32] study excluded patients with a history of VTE, which could have led to a lower risk of DVT at baseline in the included sample, thus explaining the low prevalence of DVT in this subgroup. We observed high degrees of heterogeneity in the prevalence estimates (*I*^2^ > 50%, *p* < 0.05), which could be explained by the differences in baseline risk of the patients. Some of the literature excludes patients with a history of DVT prior to admission, while a previous study showed that patients with a history of venous embolism had a significantly higher risk of reoccurring VTE after knee arthroscopy [38], suggesting that a history of DVT may influence preoperative DVT prevalence. A random-effects model was used to maintain the accuracy of the results due to the large heterogeneity between studies.

Our systematic review has several strengths. First, the studies included in this review were original studies of high quality and with a total sample size of 18,119, making it a large-scale study on the prevalence of preoperative DVT in lower extremity fractures. Second, our research provides a systematic estimation of the overall pooled prevalence of preoperative DVT in lower extremity fractures, and it further clarifies the prevalence of DVT in different subgroups to provide targeted referential suggestions for clinical work. Finally, most of the previous reviews discussed the postoperative prevalence of DVT, while our research focused on the preoperative prevalence of DVT in long bone fractures of the lower extremities, thus playing an important complementary role. The limitations of this study include the following. (i) Only nine of the studies included were cohort studies (which had the highest level of evidence), while the remaining 14 were case–control studies, which to some extent limits the level of evidence in the article. Also, there were some confounding factors in these studies, resulting in greater heterogeneity in the results. (ii) Twenty-two of the 23 articles included in this study sampled in China, and the data for the other article came from Korea. The lack of data from European and American countries could lead to poor extrapolation of our results for these populations. (iii) Given the accuracy of our results, we could not include some studies which did not distinguish DVT prevalence from VTE prevalence, which may have made the results less comprehensive. (iv) A substantial proportion (48%) of the included studies used anticoagulation before the detection of DVT, which may have resulted in an underestimate of the DVT prevalence. (v) One factor, the DVT detection time period, is not mentioned in some of the included studies, and the fracture sites could not be classified into more detailed categories, so we are unable to provide a corresponding estimate of DVT. This suggests that the timing of DVT screening, antithrombotic use and the fracture sites should be uniformly defined in future studies in order to derive a more scientifically rationalized focus for DVT screening.

This meta-analysis focused on the formation of preoperative DVT, and thus could help to lay greater emphasis on preoperative DVT screening and prevention. Existing guidelines only state that ultrasound should be routinely performed to clarify the diagnosis during the perioperative period in patients with lower extremity fractures (class IIA recommendation) [8]; they do not emphasize the importance of early preoperative DVT screening and prevention. In our study, the prevalence of preoperative DVT in lower extremity fractures was up to 24.1%. Considering that preoperative DVT may seriously affect the patient’s prognosis, high priority should be given to the screening and prevention of preoperative DVT in the management of lower extremity fractures.

## Data Availability

The datasets generated and/or analyzed during the current study are available throughout the manuscript.

## Abbreviations

DVT: Deep vein thrombosis
PTS: Post-thrombotic syndrome
PE: Pulmonary embolism
PROSPERO: International Prospective Register of Systematic Reviews
NOS: Newcastle–Ottawa scale
VTE: Venous thromboembolism
CIs: Confidence intervals
